# What Influences Adolescent Girls’ Decision-Making Regarding Contraceptive Methods Use and Childbearing? A Qualitative Exploratory Study in Rangpur District, Bangladesh

**DOI:** 10.1371/journal.pone.0157664

**Published:** 2016-06-23

**Authors:** A. S. M. Shahabuddin, Christiana Nöstlinger, Thérèse Delvaux, Malabika Sarker, Azucena Bardají, Vincent De Brouwere, Jacqueline E. W. Broerse

**Affiliations:** 1 Woman and Child Health Research Centre, Department of Public Health, Institute of Tropical Medicine, Antwerp, Belgium; 2 Unit of HIV/AIDS Policy, Department of Public Health, Institute of Tropical Medicine, Antwerp, Belgium; 3 James P Grant School of Public Health, BRAC University, Dhaka, Bangladesh; 4 ISGlobal, Barcelona Centre for International Health Research (CRESIB), Hospital Clínic-Universitat de Barcelona, Barcelona, Spain; 5 Athena Institute for Research on Innovation and Communication in Health and Life Sciences, VU University, Amsterdam, The Netherlands; Tulane University School of Public Health, UNITED STATES

## Abstract

**Background:**

Bangladesh has the highest rate of adolescent pregnancy in South Asia. Child marriage is one of the leading causes of pregnancies among adolescent girls. Although the country’s contraceptive prevalence rate is quite satisfactory, only 52% of married adolescent girls use contraceptive methods. This qualitative study is aimed at exploring the factors that influence adolescent girls’ decision-making process in relation to contraceptive methods use and childbearing.

**Methods and Results:**

We collected qualitative data from study participants living in Rangpur district, Bangladesh. We conducted 35 in-depth interviews with married adolescent girls, 4 key informant interviews, and one focus group discussion with community health workers. Adolescent girls showed very low decision-making autonomy towards contraceptive methods use and childbearing. Decisions were mainly made by either their husbands or mothers-in-law. When husbands were unemployed and financially dependent on their parents, then the mothers-in-law played most important role for contraceptive use and childbearing decisions. Lack of reproductive health knowledge, lack of negotiation and communication ability with husbands and family members, and mistrust towards contraceptive methods also appeared as influential factors against using contraception resulting in early childbearing among married adolescent girls.

**Conclusions:**

Husbands and mothers-in-law of newly married adolescent girls need to be actively involved in health interventions so that they make more informed decisions regarding contraceptive use to delay pregnancies until 20 years of age. Misunderstanding and distrust regarding contraceptives can be diminished by engaging the wider societal actors in health intervention including neighbours, and other family members.

## Introduction

The global health community considers Bangladesh as an exceptional health performer in the area of women and child health regarding family planning [1]. The country’s contraceptive prevalence rate (CPR) increased from 8% in 1975 to 62% in 2014 [2]. The public sector remains the prime source for distributing contraceptive methods for more than half of the users (52%) and government field workers are crucial in supplying the methods to about 23% of all users [3]. The country’s total fertility rate (TFR) decreased from 6.3 births per woman in 1975 to 2.3 births per woman in 2014 [2].

Despite this steady decline in fertility levels, Bangladesh has the highest rate of adolescent (aged 10–19 years) pregnancy (31%) among all the countries in South Asia, ahead of Nepal (21%) and India (21%) [2,4]. Although the country’s CPR is quite satisfactory, only 52% of married adolescent girls (aged 15–19 years) use contraceptive methods [2]. Like in many other parts of the world, women traditionally get married and have their first child during adolescence in Bangladesh [4,5]. Despite the fact that the legal age of marriage in Bangladesh is 18 years, about 66% of the women get married before the age of 18 [6]. Once a girl is married, she is usually under strong pressure to initiate childbearing by both her spouse and family and the subsequent failure to use contraceptive methods leads to childbearing among adolescents [7,8].

Pregnancies among adolescents may have detrimental health effects for both mothers and their newborns because of the higher risk of maternal complications including eclampsia, postpartum hemorrhaging, systemic infections, and preterm delivery as compared to pregnant adult women [9–11]. Babies born to adolescent mothers often suffer from low birth weight and early neonatal deaths[10–13]. Studies showed that pregnancies during adolescence can also have negative social and economic effects on the girls, their families, and communities due to school-dropout and subsequently reduced opportunities in the labor market [14–16].

Behavioral patterns on contraceptive acceptance and use differ significantly between adolescents and adult women [17,18]. Adolescent girls are less likely to use reproductive health services such as the use of contraceptive methods compared to adults [17,19]. This difference may be explained due to a lack of knowledge and/or experience with contraceptive methods as well as by a lack of decision making autonomy among adolescent girls [17–19]. In addition, low education, social conservatism, and cultural restrictions on accessing family planning methods immediately after marriage restrict the use of family planning methods for adolescent girls [5,20–22]

Despite substantial progress in the areas of maternal and child health, the issue of adolescent pregnancy remains a vital problem in Bangladesh. Several quantitative studies were conducted to identify the determinants of contraceptive use among married adolescents and adult women. Most of the studies found that socio-demographic characteristics (i.e. age, education, place of residence), income, communication with husbands, women’s involvement in social networks, and visit of healthcare workers were the main determinants of contraceptive use among women in Bangladesh [4,9,23–25].

However, until now, little qualitative work has been conducted to better understand the reasons for the high occurrence of adolescent pregnancies, such as adolescent girls’ knowledge, perceptions, and decision-making autonomy related to the use of contraceptive methods and childbearing. This study, which is part of a prospective qualitative study, aims to fill this gap and to contribute to knowledge generation in the field of adolescent pregnancy and contraceptive behavior. Our study aims at exploring the factors that influence married adolescent girls’ decision-making process in relation to contraceptive methods’ use and childbearing.

## Materials and Methods

This research was part of a larger qualitative study conducted in Rangpur district in Bangladesh. In the main study, a total of 35 married adolescent girls were followed for one year to explore their maternal healthcare-seeking behavior. The study consisted of a qualitative baseline and follow-up data collection. Here, we analyze the baseline qualitative data related to adolescent girls’ use of contraceptive methods and childbearing.

### Study population and sampling

This study was conducted in Rangpur district in the Division of Rangpur which shows the highest rate (37%) of adolescent pregnancy in Bangladesh [2]. There is no official record of pregnancies among unmarried girls since pregnancy outside of a marital bond is taboo in Bangladesh [4]. Data was collected from three rural sub-districts of Rangpur: Mithapukur, Kaunia, and Badarganj. Socio-economic patterns, cultural practices, religion (i.e. 90% Muslim population), and access to skilled maternal health services are quite similar for the people living in these three sub-districts.

Study participants were recruited purposively to cover a wider range of perspectives (i.e. depending on the research objectives) from the following groups:

- Married pregnant and non-pregnant adolescent girls aged between 12–19 years in order to explore their experiences related to contraceptive use and childbearing through in-depth interviews (IDIs). From the larger qualitative study, we purposively selected participants from various locations, i.e. sub-districts, different socio-economic and educational backgrounds, and different age-groups to achieve sufficient variation in the sample.
- Community health workers (CHWs) who had more than two years of working experience at the reproductive health project in Rangpur were recruited for a focus group discussion (FGD) to explore common practices and barriers of using contraceptive methods by the married girls.
- Representatives from the government (deputy director of family planning, Rangpur), an NGO (district level project managers of BRAC’s maternal health project), and a hospital (gynecologist from Rangpur Medical College Hospital) were interviewed as key informants because of their relevant knowledge on socio-cultural practices relating to the reproductive healthcare-seeking behavior of women in Rangpur district and their experiences in working in family planning and maternal health areas.

### Data collection

Two female research assistants (trained anthropologists experienced in conducting IDIs and FGD) collected data. The first author conducted key informant interviews (KIIs). In order to recruit married pregnant and non-pregnant adolescent girls for our study, our research team collaborated with BRAC (Bangladesh Rural Advancement Committee, a Bangladeshi NGO) staff working in Rangpur district. BRAC has been implementing a maternal health project in Rangpur district and recruited community health workers called Shasthya Shebika (SS) in every village of the Rangpur district. Each SS maintains a register that contains information about newly married couples and newly identified pregnant women, including socio-demographic information (i.e. age, duration of marriage, pregnancy etc.). We first identified our study respondents (married adolescent girls) through that register. Later, with the help of SS (or sometimes the Shasthya Kormi or program organizer) the potential participants’ homes were identified in the community. SS then introduced us to the participants. Afterwards, we obtained informed consent from them before starting the interview. Table 1 shows the types of respondents and data collection methods (Table 1).

**Table 1:** Data collection methods and respondents.

In-depth interviews (IDIs)	Characteristics of the respondents	Pregnant adolescent girls (n = 25)	Non-pregnant adolescent girls (n = 10)
	**Respondents’ age**		
	Younger than 18 years	20	7
	18–19 years	5	3
	**Year of marriage**		
	Within last 1 year	3	10
	>1 year	22	
	**Sub-district**		
	Kaunia	9	2
	Badarganj	6	7
	Mithapukur	10	1
	**Religion**		
	Islam	23	9
	Hinduism	2	1
	**Type of consent provided**		
	Written consent by adolescent girls	4 (among those aged 18–19 years)	3 (among those aged 18–19 years)
	Verbal consent by adolescent girls	1 (among those aged 18–19 years)	0
	Written Consent from guardians	18	6
	Verbal consent from guardians	7	4
**Key informant interviews (KIIs)**	**NGO representatives**	**Government representative**	**Health providers**
	2 NGO representatives (more than 5 years working experience in maternal health projects in Rangpur division)	1 deputy director of family planning in Rangpur district	1 gynecologist of Rangpur Medical College hospital (RMCH) (more than 3 years working experiences at RMCH)
**Focus group discussion (FGD)**	**Community health workers**		
	6 CHWs (Shasthya Shebika of BRAC maternal health project) with more than 3 years of working experiences		

Interviews and FGD were conducted using thematic topic guides covering issues related to: the socio-demographic characteristics of the respondents, adolescent girls’ knowledge, perceptions, and practice related to contraceptive methods’ use and childbearing; girls’ decision making autonomy regarding contraceptive use and childbearing; access and availability of contraceptives; and socio-cultural beliefs and practice related to contraceptive methods’ use and pregnancy.

The topic guides for IDI and KII were pretested to determine feasibility and to refine the questions prior to final data collection. We pretested the topic guide for both pregnant and non-pregnant married adolescent girls. Pretesting was done with two pregnant and two non-pregnant married adolescent girls from Rangpur Sadar Upazila in Rangpur district. The interviewers were trained on primary and final topic guides. Topic guide for KII was pretested with a public health professional working in the area of maternal health.

The topic guides were developed in English and translated into Bangla, before pre-testing. Due to logistical issues (e.g. time constraint, difficulties to find CHWs to gather in a place) the FGD topic guide could not be piloted. While collecting the data, questions were adjusted and probed to investigate in more detail about participants’ experiences and their perceptions.

### Data analysis

Data collected from IDIs, KIIs, and FGD were audio-taped and transcribed verbatim in Bangla. Research assistants and the first author took extensive field notes. Bangla transcripts were read several times to get acquainted with raw data and checked at random for accuracy by the first author and adapted when necessary. Professional translators translated the Bangla transcripts into English. The first author (a Bangladeshi speaking Bangla and English) cross-checked all English translated transcripts with Bangla transcripts (also going back to the audio files whenever necessary) to ensure the quality of translation.

We used a thematic analysis approach for analyzing the qualitative data [26]. First, we generated initial codes and coded the text sections of all the transcripts through repeated reading. Then we identified relevant themes in line with the codes and labeled the themes afterwards. We considered each theme as an influencing ‘factor’ on contraceptive methods use and childbearing decisions. In order to increase the validity of the findings, the first and second authors independently coded transcripts and consensus was obtained after discussion on controversial issues. MAXQDA 11 software was used for structuring and analysis of the data.

### Ethical considerations

The research protocol was approved by the Institutional Review Board (IRB) of the Institute of Tropical Medicine (ITM) in Antwerp. Ethical approval was also obtained from the Ethical Review Committee (ERC) of the James P. Grant School of Public Health at BRAC University, Bangladesh. Written informed consent was sought from all the participants. However, because of the cultural issue (i.e. respondent felt uncomfortable to provide signature) and illiteracy of the study participants, verbal consent was taken from few of the respondents. Written informed consent was documented through a signature on a ‘participant information sheet and informed consent’ form and verbal informed consent was documented via audio recording. Respondents aged below 18 provided assent, while written consent was sought from their legal guardians/husbands. Written informed consent was obtained from all FGD participants. Confidentiality was strictly maintained: only the researchers had access to the data and no personal identifying information was kept that could possibly link to the respondents after completion of the analysis.

## Results

### Major findings

- Married adolescent girls in Rangpur district, Bangladesh had limited knowledge of proper use of contraceptive methods.
- Decision making autonomy towards contraceptive methods use and childbearing was very low among adolescent girls.
- Husband and mother-in-law acted as the main decision makers regarding contraceptive methods use and childbearing. Expectations of bearing a child or grandson led to an adolescent girl becoming pregnant just after marriage.
- Mistrust and fear regarding the quality and use of contraceptive methods from sisters-in-laws and neighbors further restricted adolescent girls to use of contraceptive methods after marriage.
- Husbands and mothers-in-law should be sensitized about the importance of adolescent girls’ autonomy in decision-making regarding their sexual and reproductive health.
- Wider social actors should be involved in sexual and reproductive health interventions targeting this group and support girls’ autonomy in decision-taking in order to decrease the rates of adolescent pregnancies in rural Bangladesh.

### Adolescent girls’ background characteristics

#### Socio-demographic characteristics of the adolescent girls

Table 2 represents the background characteristics of the 35 married adolescent girls who participated in the research. Among them, 25 were pregnant and 10 were non-pregnant adolescents. The mean age of the respondents was 16 years. Eleven of the respondents were between 12–15 years old, 24 of them were in the age range 16–19 years (Table 2).

**Table 2:** Background characteristics of study participants (n = 35).

Characteristics	*N*
**Age (in years)**	
12–15 years	***11***
16–19 years	***24***
**Education of the respondent**	
No formal education	***1***
Primary school or less	***11***
Secondary school (any)	***22***
Higher than secondary	***1***
**Continuation of education after marriage**	
Education continued	***3***
Dropout of school	***32***
**Occupation of the respondent**	
Unemployed (housewife)	***32***
Student	***3***
Employed	***0***
**Age of the husband**	
<20 years	***13***
20–30 years	***18***
>30 years	***4***
**Occupation of the husband**	
Unemployed	***2***
Agriculture/farming	***10***
Business (i.e. owner of a shop)	***16***
Others (i.e. rickshaw puller, daily laborer)	***7***
Casual job	***0***
**Education of the husband**	
No formal education	***4***
Primary school or less	***11***
Secondary school	***18***
Higher than secondary	***2***
**Pregnancy status**	
Currently pregnant	***25***
Non-pregnant (recently married)	***10***
**Pregnancy intentions (n = 25)**	
Pregnancy planned	***16***
Pregnancy unplanned	***9***

#### School drop-out

Among the 35 adolescent girls interviewed, seven had left school before marriage and 25 girls after marriage. Only three girls were studying at a secondary level during the time of interview (Table 1). Several reasons were mentioned for dropping out from school including: unwillingness of the family members (e.g. husbands and parents-in-law) to send married girls to school, lack of financial support from the girls’ own parents, parents-in-law and husbands, the workload of a newly married girl in a new family, and loss of interest in study after marriage. This is illustrated by a quote from a 17-year old pregnant adolescent (IDI): “*If I study then who will do the work*? *I don’t have scope for the education now*. *I do cook and other things by myself*, *isn’t this the problem to study further*? *That’s why my husband said no need to go to school”*.

Adolescent girls’ husbands also had low education levels. Among them, two had higher secondary education levels, while four of them did not have any formal education.

#### Marriage during adolescence

Most of the adolescent girls reported that their marriage had been arranged by family members, in most of the cases without their consent. Four girls were informed about their marriage just before it was arranged. A 15-year old pregnant adolescent girl (IDI) said: *“I was 14 and wanted to continue my education*.…*but my parents did not inform me about my marriage*. *One day*, *one of my grandfathers brought me here with a motorcycle and asked me to sign a paper and*, *I signed that*. *Then after coming back to home*, *I came to know that it was a registration for marriage*. *After some days they came to put on nose pin then I understood that I am married”*. A few respondents mentioned that they were willing to get married because they had nothing to do at home. In addition, two girls mentioned that they eloped and got married.

#### Adolescent girls’ decision making

**Fig 1:**
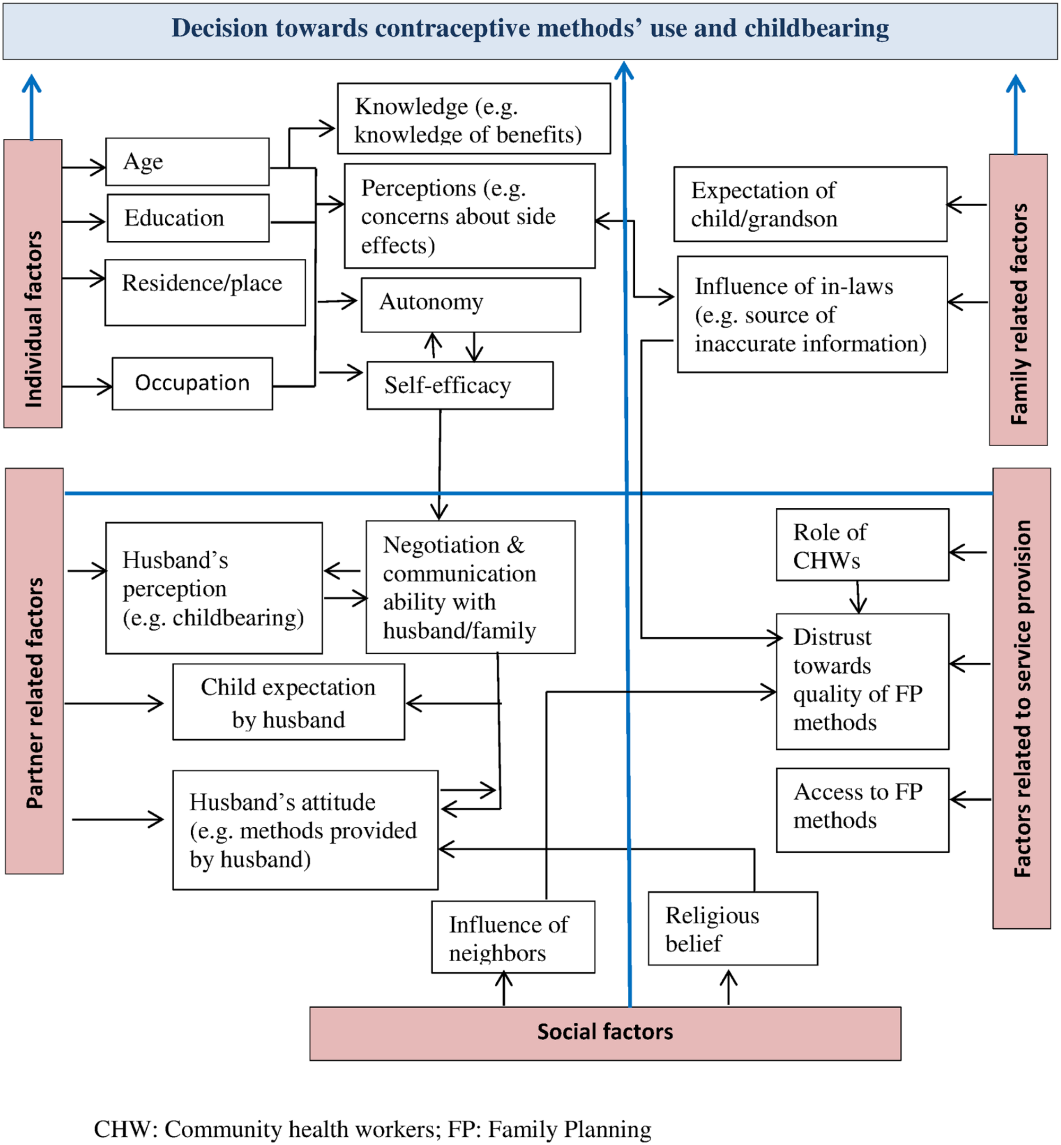
Emerging codes and factors.

Five main factors emerged which influenced adolescent girls’ decision-making on contraceptive methods and childbearing: *individual factors*, *partner related factors*, *family related factors*, *social factors and factors related to service provision (*Fig 1*)*.

### Individual factors

#### Knowledge and perception about contraceptive methods and childbearing

Most of the adolescent girls had limited knowledge about modern contraceptive methods and the correct use (i.e. such as dosing, timing and effects) of the methods. A few girls even said that they came to know about contraceptive methods after the termination of their first pregnancy or after having their first child, as illustrated by the following quotes: *“I didn’t understand that much then that’s why I did not use any method*. *I aborted (natural) my first child*. *Now*, *I understand*. *I used it for a while*, *and then again stopped to have a baby”—(17-year old pregnant adolescent*, *IDI)*.

Moreover, contraceptive methods were often perceived as harmful to the body and a cause of infertility which restricted their use. An 18-year old pregnant adolescent girl (IDI) described the following: *“My husband told me to delay to take baby but I thought it (pills) will give me an inflammation in my belly button if I have it*! *People also said that if you eat [take] pills you will not have baby in the future”*.

In terms of knowledge about childbearing, most of the adolescent girls did not have any concrete information about the consequences of early pregnancies but somehow understood that being pregnant at young age carried risks for mother and child.

*“I heard…there might be convulsion due to early age pregnancy*. *It is very risky and might also cause visionary problem*, *will cause nausea and can also store water in legs and in hands (18-year age pregnant adolescent girl, IDI)*. However, their concern of being pregnant at an early age did not lead to a delay in pregnancies.

#### Autonomy and negotiation skills with partners and family

Almost all the adolescent girls had very little decision making autonomy. Decisions regarding contraceptive methods’ use or childbearing were mainly made by others (mainly husbands or mothers-in-law) and not by the adolescent girls themselves. Few girls could negotiate or communicate with their husbands on this issue, and they had to follow their husband’s or other family members’ decision on whether or not to use contraceptive methods or to have a child.

*“He (husband) told me to take a baby*. *What could I say then*? *My mother-in-law also asked me to have a baby as there is no baby at my in-laws’ house”- (18-year old pregnant adolescent*, *IDI)*

Shyness was mentioned as one of the reasons which led adolescent girls not to communicate with their partners and family members about use of contraceptives and childbearing. One of the CHWs participating in the FGD echoed that: “*Teenage girls can’t share and also can’t discuss everything because of shyness”*

The results showed that individual factors such as self-efficacy in decision making were largely influenced by contextual conditions, for instance by the financial dependency on the husband. The latter was mentioned as one of the main reasons for low decision making autonomy. *“A woman can take her own decision only when she is self-dependent*. *Even if a lady is postgraduate but remains as a house wife*, *her decision won’t be countable”–(Community health worker*, *FGD)*

### Partner related factors

Our study found that the husband (often, the household head) played the most important role in terms of contraceptive methods’ use and childbearing of a newly married girl. When husbands were financially stable or working, they mainly made the decision of whether or not to use contraceptive methods and thus, ultimately resulting in pregnancy. A 14-year old pregnant adolescent mentioned in IDI: “*I wanted to take pills consecutively for two to three years*. *But my husband did not allow me to take the pills*. *He was against of using any method*”. In most of the cases, girls had to depend on their husbands for obtaining access to oral contraceptives, although they could receive or buy contraceptives from CHWs. Sometimes, husbands refused to provide their wives with the pills, as illustrated by the following quote. *“I didn’t want to have the baby*. *But what could I do if he (husband) didn’t bring me pills*!*”- (18-year old pregnant adolescent*, *IDI)*.

In some cases, husbands were supportive and influenced their wives to use a contraceptive method to delay pregnancy: *“Umm*.*…my husband told me that now I am very young*. *Then he told me that*, *“You are studying*.*…if you take a baby now then your study will be hampered*. *Thus*, *you couldn’t continue your education*. *How would you maintain your baby and you together*?*”- (16-year old secondary school going non-pregnant adolescent*, *IDI)*

### Family related factors

Mothers-in-law played a very important role in the adolescent girls’ decision-making process on contraceptive use and childbearing. Their decisions were very influential especially when the husbands of married adolescent girls were financially dependent upon their parents. Evidently, mothers-in-law’s expectation to have a grandson played a key role. A 15-year old pregnant girl mentioned (IDI): *“I told my husband not to have the baby now as many things are needed for it*. *Once*, *my mother-in-law came and told me if she (mother-in-law) dies someday without seeing the face of grandchild [especially grandson]*?*”*

Also during the KII, the role of the mother-in-law was mentioned as an influencing factor: “*If a married girl does not conceive a child within 3 years of marriage then the mother-in-law will accuse or put blame on her for the sake of grandchild [grandson]*. *This is what happens usually”—(NGO representative*, *KII)*.

Sisters-in-law (the husband’s own sister or cousin sister) played an important role in terms of providing information about reproductive health issues including types and use of family planning methods. Sometimes, adolescent girls received inaccurate information from sisters-in-law who forbade them to use contraceptive methods as shown by the following example: *“My sister in-law told me that if I have pills for long*, *I might have to do caesarean section for childbirth”- (18-year old pregnant adolescent*, *IDI)*

### Social factors

Social factors such as the influence of neighbors, socio-cultural beliefs and practices, religious belief, and myths also shaped the decision-making process on contraceptive use and childbearing to various degrees. The influence of neighbors on decisions making related to contraceptive use and childbearing was mixed. A few respondents mentioned that some neighbors had scared them by talking about the negative effects of using contraceptive methods, for example: *“My neighbors came to know that I took injection (a hormonal contraceptive method) as my mother-in-law told them*. *They scared me saying that I won’t be able to conceive a baby due to the injection and so other things [using injection at young age]”-(15-year old pregnant adolescent*, *IDI)*. To some extent, religious beliefs played a role in the timing of childbirth. A pregnant adolescent girl said that her husband forbade her to take any birth control as he wanted the baby during ‘Ramadan’, which is considered the most holy month by Muslims. Moreover, adolescent girls who belonged to families adhering strictly to religious rules were less likely to use family planning methods.

### Factors related to service provision

Almost all adolescent girls mentioned about the availability of community health workers in providing contraceptive methods in the community, mainly recruited by the BRAC maternal health project.

Government health workers were rarely seen in the communities. However, several respondents questioned and doubted the quality of the contraceptive options provided by the government. One respondent mentioned that the pills from the government were not good enough and that she preferred pills from shops, as illustrated by the following quote “*Yes*, *I took pill*. *Now I don’t take these pills from the government as a sister told me that if I have the government pills then I could not conceive a baby*.*… there will be problems to have a baby as you are young*. *She told me to buy pills from the shops and to take it”-(16-year age non-pregnant adolescent girl*, *IDI)*.

## Discussion

This study revealed that factors situated on different levels (i.e. individual, partner, family and social) impacted adolescent girls’ decision-making autonomy, which influenced actual contraceptive behaviour and thus led to child-bearing. These factors emerged largely similarly from the various data collection sources. They were intertwined and influenced each other (as can be seen in Fig 1). Lack of adequate reproductive health knowledge, low decision-making autonomy, lack of negotiation and communication with husbands and members of the in-law’s family, the expectation of a child by husbands, the expectation of grandsons by mothers-in-law, and mistrust towards contraceptive methods appeared as major factors that influenced adolescent girls’ decision not to use any contraceptive methods thus, resulting in childbearing.

Our findings show that a small proportion of adolescents married girls who were still at school are in line with other studies [15,27]. In addition, literature shows that dropping out from school after marriage restricts adolescent girls’ broadening of knowledge about reproductive health [15]. Also in line with the findings in a systematic review, this study showed that limited knowledge about reproductive health including contraceptive use and safe motherhood may lead to unplanned pregnancies [28]. In terms of decision-making autonomy, like other studies conducted in Bangladesh and Kenya, this study also found that adolescent girls had very low decision-making autonomy regarding contraceptive methods’ use and childbearing [24,29]. A low level of education, unemployment, and financial dependency on husbands or parents-in-law put many adolescent girls in a situation where, often, their voices are not taken into account by others [30,31]. In addition, their age at marriage correlates with the girl’s position in a family. When a girl is married during adolescence, she is automatically put in a situation where she is less empowered in the social structure [30].

A study conducted in Bangladesh found that the husband played an important role in making decisions regarding contraceptive use and childbearing which also supports the findings of this study [24]. Another study also showed the influence that mothers-in-law have on contraceptive use and childbearing in the decision-making process [32,33]. Two aspects made husbands and mothers-in-law influential in this process. Firstly, in the context of rural Bangladesh, men are usually the wage earners, lead the household, and ultimately make most of the household decisions regarding health care [18]. Secondly, family planning is still considered a matter for women, hence mothers-in-law are perceived as the most experienced people in this matter [24]. When husbands did not have a job or income, then mothers-in-law became more influential in the household. Regardless of the income status of husbands, the key role of mothers-in-law is well understood from different studies conducted in Bangladesh and neighboring country Pakistan [24,23].

In line with the results of previous studies conducted in Bangladesh and its neighbouring country Nepal, this study showed that a lack of communication and negotiation ability with the husband negatively affects informed decision-making on contraceptive use and childbearing [7,33,34]. Because of the age and power differentials between wives and husbands, often a newly married adolescent girl hesitates or refrains from talking to her husband about contraceptive methods which limits inter-spousal communication.

After marriage, mainly sisters-in-law (cousin-sisters’ of husbands, husbands’ own sisters or wives of husband’s brothers) and neighbors (mainly married girls living in the same areas) become the primary sources of information relating to reproductive health including contraceptives [35]. This study found that adolescent girls often received incorrect or misleading information from neighbours and sisters-in-law which led to mistrust about the use of contraceptive methods. Mistrust and misleading information also scared women and influenced them not to use contraceptive methods before having the first child [33].

Studies showed that visit of community health workers positively affected the use of contraceptive methods [36,37]. Government of Bangladesh has deployed CHWs (e.g. family welfare assistants and female health assistants) in almost every village in Bangladesh to provide door to door family planning services. However, this study showed that adolescent girls were more in contact with CHWs (e.g. SSs) deployed by the BRAC and CHWs deployed by the government were rarely in touch with the adolescent girls. Less frequent contact of government CHWs with adolescent girls might be the reason of existing mistrust about the quality of contraceptive methods provided by the government.

### Limitations of the study

Because of the rural study setting, the results might not be generalizable to urban settings in Bangladesh where adolescent girls tend to have different socio-economic backgrounds. We collected data from 35 married adolescents with different profiles and other stakeholders in order to increase the validity of the information. However, we believe that the information we gathered was rich enough to present a realistic scenario of the factors influencing adolescent girls’ decision-making process on contraceptive use and childbearing. In addition, a potential limitation was trepidation on the part of the adolescent respondents, who were not used to being interviewed about these sensitive subjects and also were not accustomed to sharing their opinion and personal experiences in general.

### Programmatic implications

In order to increase the uptake of contraceptives for delaying pregnancies among married adolescent girls, the results of this study emphasize that husbands and mothers-in-law of newly married adolescent girls need to be actively involved in health interventions so that they can make more informed decisions regarding contraceptive use to delay pregnancies up to 20 years [38].

Misunderstanding and mistrust regarding contraceptives can be addressed by engaging the wider community or “societal actors” when it comes to health intervention by including other family members (i.e. sisters-in-law) of the adolescent girls and neighbours. Adolescent girls, family members, and the community members at large should have correct information about the benefits of using contraceptive methods and risks of adolescent pregnancies. The community’s elite including religious leaders, school teachers, community representatives, and Kazi (who conducts marriage and does registration) should also be involved in the intervention as they are well respected by the community (suggested by KII participants).

Clearly, interventions that focus both on the continuation of the girls’ education and creating options for generating income (i.e. life skills training) are also worthwhile towards increasing their reproductive health knowledge and in strengthening their decision-making autonomy.

## References

[1] Chowdhury AMR, Bhuiya A, Chowdhury ME, Rasheed S, Hussain Z, Chen LC. The Bangladesh paradox: Exceptional health achievement despite economic poverty. Lancet. 2013;382: 1734–1745. 10.1016/S0140-6736(13)62148-0 24268002

[2] National Institute of Population Research and Training (NIPORT), Mitra and Associates and II. Bangladesh Demographic and Health Survey 2014: Key Indicators. Dhaka, Bangladesh, and Rockville, Maryland, USA; 2015.

[3] National Institute of Population Research and Training (NIPORT). Bangladesh Demography and Health Survey 2011. Dhaka, Bangladesh, and Rockville, Maryland, USA; 2012.

[4] Sayem AM, Nury ATM. Factors associated with teenage marital pregnancy among Bangladeshi women. Reproductive Health. 2011 p. 16 10.1186/1742-4755-8-16 21599904 PMC3187734

[5] Santos KA. Teenage pregnancy contextualized: understanding reproductive intentions in a Brazilian shantytown. Cad Saude Publica. 2012;28: 655–664. 10.1590/S0102-311X2012000400005 22488311

[6] United Nations Children’s Fund. Progress and prospects [Internet]. Ending Child Marriage: Progress and prospects, UNICEF. 2014 10.1016/j.landurbplan.2012.01.010

[7] Shahidul Islam M, Shafiul Alam M, Mahedi Hasan M. Inter-spousal communication on family planning and its effect on contraceptive use and method choice in Bangladesh. Asian Soc Sci. 2014;10: 189–201. 10.5539/ass.v10n2p189

[8] Kamal SMM, Hassan CH, Alam GM, Ying Y. Child Marriage in Bangladesh: Trends and Determinants. J Biosoc Sci. 2014; 1–20. 10.1017/S0021932013000746 24480489

[9] Chen X-K, Wen SW, Fleming N, Demissie K, Rhoads GG, Walker M. Teenage pregnancy and adverse birth outcomes: a large population based retrospective cohort study. Int J Epidemiol. 2007;36: 368–73. 10.1093/ije/dyl284 17213208

[10] Conde-Agudelo A, Belizán JM, Lammers C. Maternal-perinatal morbidity and mortality associated with adolescent pregnancy in Latin America: Cross-sectional study. American Journal of Obstetrics and Gynecology. 2005 pp. 342–349. 10.1016/j.ajog.2004.10.593 15695970

[11] Ganchimeg T, Ota E, Morisaki N, Laopaiboon M, Lumbiganon P, Zhang J, et al Pregnancy and childbirth outcomes among adolescent mothers: a World Health Organization multicountry study. BJOG. 2014;121 Suppl: 40–48. 10.1111/1471-0528.12630 24641534

[12] Chen XK, Wen SW, Fleming N, Yang Q, Walker MC. Increased risks of neonatal and postneonatal mortality associated with teenage pregnancy had different explanations. J Clin Epidemiol. 2008;61: 688–694. 10.1016/j.jclinepi.2007.08.009 18538263

[13] Mukhopadhyay P, Chaudhuri RN, Paul B. Hospital-based perinatal outcomes and complications in teenage pregnancy in India. J Heal Popul Nutr. 2010;28: 494–500. 10.3329/jhpn.v28i5.6158 PMC2963772 20941901

[14] Kamal SMM, Hassan CH. Child Marriage and Its Association With Adverse Reproductive Outcomes for Women in Bangladesh. Asia Pac J Public Health. 2013; 10.1177/1010539513503868 24097938

[15] Lloyd CB, Mensch BS. Marriage and childbirth as factors in dropping out from school: an analysis of DHS data from sub-Saharan Africa. Popul Stud. 2008;62: 13. 10.1080/00324720701810840 18278669

[16] World Health Organization. Adolescent pregnancy. Fact sheet N°364. Geneva; 2012.

[17] Blanc AK, Tsui AO, Croft TN, Trevitt JL. Patterns and trends in adolescents’ contraceptive use and discontinuation in developing countries and comparisons with adult women. Int Perspect Sex Reprod Health. 2009;35: 63–71. 10.1363/3506309 19620090

[18] Haque SE, Rahman M, Mostofa MG, Zahan MS. Reproductive Health Care Utilization among Young Mothers in Bangladesh: Does Autonomy Matter? Women’s Heal Issues. 2012;22 10.1016/j.whi.2011.08.004 21968029

[19] Asaduzzaman M, Khan MHR. Identifying Potential Factors of Childbearing in Bangladesh. Asian Soc Sci. 2009;5: 147–154.

[20] Adams MK, Salazar E, Lundgren R. Tell them you are planning for the future: Gender norms and family planning among adolescents in northern Uganda. Int J Gynecol Obstet. 2013;123 10.1016/j.ijgo.2013.07.004 23992625

[21] Gayen K, Raeside R. Communication and contraception in rural Bangladesh. Healthc Q. 2006;9: 110–122. Available: http://www.ncbi.nlm.nih.gov/sites/entrez?Db=pubmed&DbFrom=pubmed&Cmd=Link&LinkName=pubmed_pubmed&LinkReadableName=Related-Articles&IdsFromResult=17076385&ordinalpos=3&itool=EntrezSystem2.PEntrez.Pubmed.Pubmed_ResultsPanel.Pubmed_RVDocSum\nhttp://www.ncbi. 17076385

[22] Gayen K, Raeside R. Social networks and contraception practice of women in rural Bangladesh. Soc Sci Med. 2010;71: 1584–1592. 10.1016/j.socscimed.2010.08.002 20869146

[23] Kadir MM, Fikree FF, Khan A, Sajan F. Do mothers-in-law matter? Family dynamics and fertility decision-making in urban squatter settlements of Karachi, Pakistan. J Biosoc Sci. 2003;35: 545–558. 10.1017/S0021932003005984 14621251

[24] Kamal SM. Childbearing and the use of contraceptive methods among married adolescents in Bangladesh. Eur J Contracept Reprod Heal Care. 2012;17: 144–154. 10.3109/13625187.2011.646014 22242676

[25] Kamal SMM, Islam MA. Contraceptive use: socioeconomic correlates and method choices in rural Bangladesh. Asia Pac J Public Health. 2010;22: 436–50. 10.1177/1010539510370780 20659903

[26] Braun V, Clarke V. Using thematic analysis in psychology. Qual Res Psychol. 2006;3: 77–101. 10.1191/1478088706qp063oa

[27] Grant MJ, Hallman KK. Pregnancy-related school dropout and prior school performance in KwaZulu-Natal, South Africa. Studies in Family Planning. 2008 pp. 369–382. 10.1111/j.1728-4465.2008.00181.x 19248721

[28] Williamson LM, Parkes A, Wight D, Petticrew M, Hart GJ. Limits to modern contraceptive use among young women in developing countries: a systematic review of qualitative research. Reprod Health. 2009;6: 3 10.1186/1742-4755-6-3 19228420 PMC2652437

[29] Ochako R, Mbondo M, Aloo S, Kaimenyi S, Thompson R, Temmerman M, et al Barriers to modern contraceptive methods uptake among young women in Kenya: a qualitative study. BMC Public Health. 2015;15: 118 10.1186/s12889-015-1483-1 25884675 PMC4336491

[30] Deb S, Kabir A, Kawsar L. Women’s Empowerment and Regional Variation of Contraceptive Norms in Bangladesh. Int Q Community Heal Educ. 2011;31: 401–410. 10.2190/IQ.31.4.g 22192945

[31] Hameed W, Azmat SK, Ali M, Sheikh MI, Abbas G, Temmerman M, et al Women’s empowerment and contraceptive use: The role of independent versus couples' decision-making, from a lower middle income country perspective. PLoS One. 2014;9 10.1371/journal.pone.0104633 PMC4131908 25119727

[32] Schuler SR, Rottach E. Women’s Empowerment across Generations in Bangladesh. J Dev Stud. 2010;46: 379–396. 10.1080/00220380903318095 20847904 PMC2938081

[33] Rahman A, Rahman M, Siddiqui MR, Zaman JA. Contraceptive practice of married women: Experience from a rural community of Bangladesh. J Med. 2014;15: 9–13. 10.3329/jom.v15i1.19852

[34] Yue K, O’Donnell C, Sparks PL. The effect of spousal communication on contraceptive use in Central Terai, Nepal. Patient Educ Couns. 2010;81: 402–408. 10.1016/j.pec.2010.07.018 20719462

[35] Khan NR, Sadia J. Prevalence of contraceptive use among married women of reproductive age groups in a rural area of Bangladesh. J Dhaka Med Coll. 2014;23: 7–13.

[36] Kabir H, Saha NC, Oliveras E, Gazi R. Association of programmatic factors with low contraceptive prevalence rates in a rural area of Bangladesh. Reprod Health. 2013;10: 31 10.1186/1742-4755-10-31 23782912 PMC3689600

[37] Hossain MB, Phillips JF. The impact of outreach on the continuity of contraceptive use in rural Bangladesh. Stud Fam Plann. 2012;27: 98–106. Available: http://www.ncbi.nlm.nih.gov/pubmed/8714307. 8714307

[38] Shattuck D, Kerner B, Gilles K, Hartmann M, Ng’ombe T, Guest G. Encouraging contraceptive uptake by motivating men to communicate about family planning: The Malawi Male Motivator project. Am J Public Health. 2011;101: 1089–1095. 10.2105/AJPH.2010.300091 21493931 PMC3093271

